# From bag-of-genes to bag-of-genomes: metabolic modelling of communities in the era of metagenome-assembled genomes

**DOI:** 10.1016/j.csbj.2020.06.028

**Published:** 2020-06-25

**Authors:** Clémence Frioux, Dipali Singh, Tamas Korcsmaros, Falk Hildebrand

**Affiliations:** a Inria, CNRS, INRAE Bordeaux, France; b Gut Microbes and Health, Quadram Institute Bioscience, Norwich, Norfolk, UK; c Microbes in the Food Chain, Quadram Institute Bioscience, Norwich, Norfolk, UK; d Digital Biology, Earlham Institute, Norwich, Norfolk, UK

**Keywords:** Bioinformatics, Metagenomics, Systems biology, Microbiota, Metabolic modelling, Metagenomic-assembled genomes, Gene Functions

## Abstract

Metagenomic sequencing of complete microbial communities has greatly enhanced our understanding of the taxonomic composition of microbiotas. This has led to breakthrough developments in bioinformatic disciplines such as assembly, gene clustering, metagenomic binning of species genomes and the discovery of an incredible, so far undiscovered, taxonomic diversity. However, functional annotations and estimating metabolic processes from single species – or communities – is still challenging. Earlier approaches relied mostly on inferring the presence of key enzymes for metabolic pathways in the whole metagenome, ignoring the genomic context of such enzymes, resulting in the ‘bag-of-genes’ approach to estimate functional capacities of microbiotas.

Here, we review recent developments in metagenomic bioinformatics, with a special focus on emerging technologies to simulate and estimate metabolic information, that can be derived from metagenomic assembled genomes. Genome-scale metabolic models can be used to model the emergent properties of microbial consortia and whole communities, and the progress in this area is reviewed. While this subfield of metagenomics is still in its infancy, it is becoming evident that there is a dire need for further bioinformatic tools to address the complex combinatorial problems in modelling the metabolism of large communities as a ‘bag-of-genomes’.

## 1 Introduction

Microbiotas are assemblies of microorganisms from a defined environment [1]. These organisms, their genomes, and their habitat form a microbiome. Microbiotas, especially host-associated microbiotas, are at the core of a highly dynamic field of research dedicated to understanding relationships across microorganisms or between microorganisms and their hosts. One example in the context of human health is the gastrointestinal-associated microbiome, which has been associated with a number of diseases [2], [3], [4]. Microbiomes have applications far beyond human health and are also scrutinised in the soil [5] or in association to plants [6]. Studying microbiomes comes with challenges when compared to studying individual organisms. Previous systems biology approaches [7] aiming to study the cell at the systems-level have to be extended at the ecosystem scale as the functioning and behaviour of a given organism is highly dependent on the interactions it harbours with others.

**Fig. 1:**
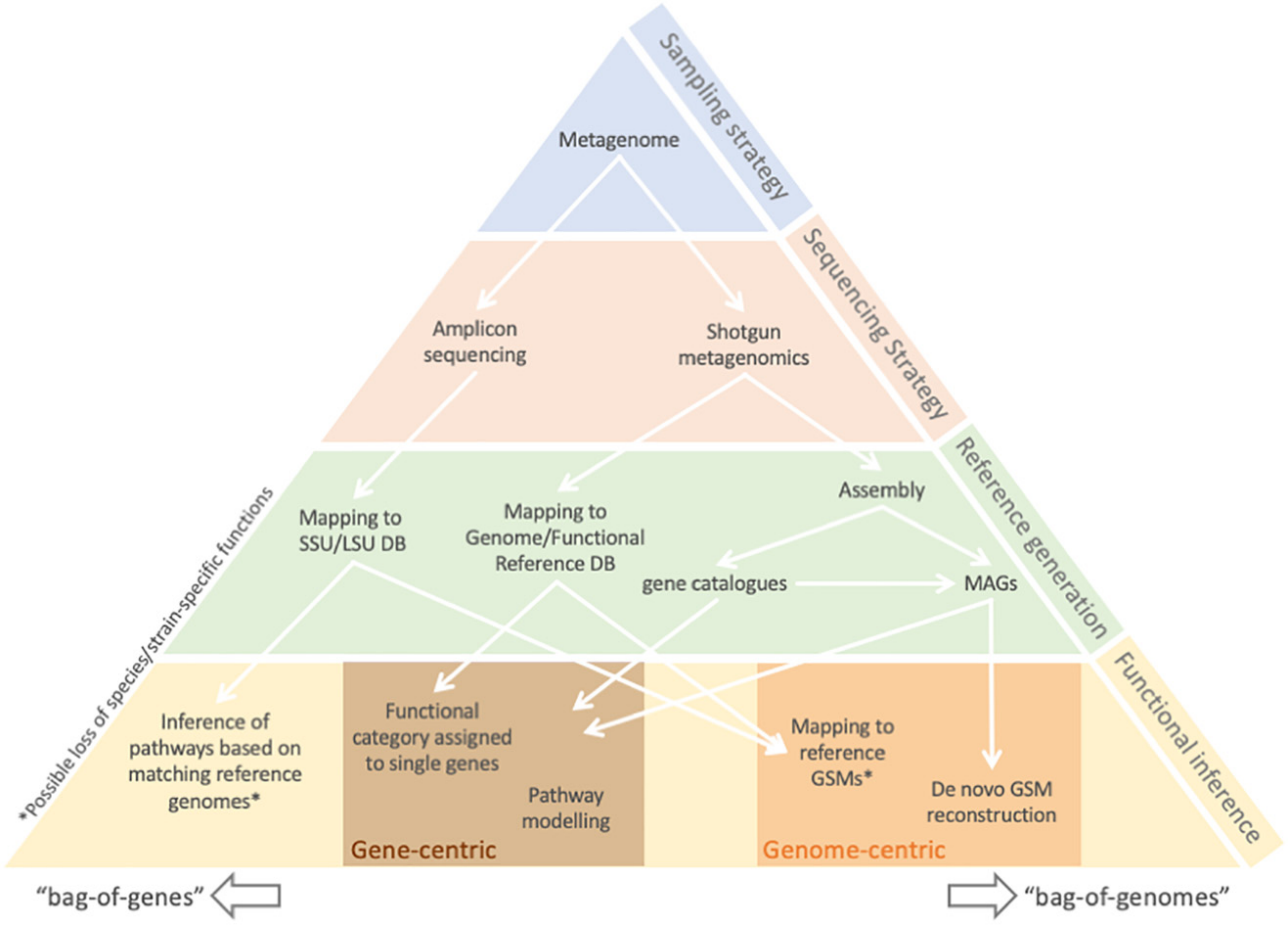
Overview of strategies for functional inference using metagenomics. SSU: Small Sub-Unit. LSU: Large Sub-Unit. DB: database. MAG: Metagenome-Assembled Genome. GSM: Genome-scale Metabolic Model.

A metagenome is defined as the genomes of all microorganisms in a given sample, that are sequenced together [8] – essentially a ‘*bag-of-genes'*. When studying microbiomes via metagenomics, usually two primary objectives have to be addressed: i) the identification of the microorganisms, ideally with quantitative information on their occurrences, and ii) the characterisation of the functions and roles they harbour within that community. Several bioinformatics strategies for the treatment of microbiota-derived sequencing data can be considered, varying in the nature and treatment of sequences and in the inference of functions (Fig. 1). Metagenomics is the discipline aiming at addressing the first objective of identifying microorganisms, through the sequencing and assembly of individual genomes derived from community samples. Traditionally, most algorithmic and software work has focused on Bacteria, while Eukaryotes and Phages/Viruses are often overlooked. Obtaining, mostly for bacteria, metagenome-assembled genomes (MAGs) of good quality is an active research area with these MAGs constituting the sole source of information associated with many non-culturable microorganisms for which no reference genome is available. The second objective dedicated to functional characterisation is classically addressed through mapping genes, or gene fragments, to functional reference databases, considering the community as a ‘*bag-of-genes*’ [9]. However, more modern approaches use MAGs, acknowledging the community consisting of single, isolated, metabolically active units (microbial species), what we termed the ‘*bag-of-genomes*’ approach.

This evolution in the field of metagenomics enables the consideration of new methods for the inference of functions in microbiotas. Classical approaches aimed at assigning functional categories and pathways to genes [10] or inferring functions for taxonomic units using close reference genomes [11] are being extended by metabolic modelling. For individual organisms, genome-scale metabolic networks and models (GSMs) are the current state-of-the-art approach for understanding the metabolism and functions carried by a species. The latter can be achieved by applying a variety of formalisms and numerical models to the networks. The field has evolved to the study of interacting organisms and communities. In this direction, MAGs can be used to build GSMs, which in turn serve as hubs in an ecosystem-level functional network. Because this approach is relatively novel and technically challenging, we describe in this mini-review the current state-of-the-art approaches for MAGs reconstruction in metagenomes and metabolic modelling for communities. We discuss how MAGs can be useful to better understand the metabolism of microbial communities, and what challenges remain in the use of ‘*bag-of-genomes*' approaches to successfully model and compare ecosystem functional predictions and the role of single hubs within these.

## 2 Metagenomic approaches to capture the diversity of microbiotas

### 2.1 Taxonomic composition of microbiomes – A historical perspective

The exploration of microbial communities with high-throughput, cost-effective and precise techniques was enabled with the advent of next generation sequencing, using either a) amplicon sequencing (also referred to as metabarcoding or metataxonomics) or b) metagenomics [12]. Metagenomics is required to obtain gene sequences that can be functionally annotated, but for completeness both approaches are briefly discussed:

#### 2.1.1 Amplicon sequencing

Amplicon sequencing is a technology closely related to metagenomics, relying on sequencing amplified gene regions to identify community compositions of samples. Specific PCR primers for different taxonomic groups are used to amplify taxonomically informative genome regions. These are typically 16S rRNA (ribosomal RNA) gene for Bacteria and Archaea [13], the 18S rRNA gene for Eukaryotes as a group and ITS (internal transcribed spacers) regions for the exploration of micro-fungi [14].

It is widely utilised due to the ease to use workflows, e.g. QIIME2, mothur and LotuS [15], [16], [17], and cost-effectiveness, as a small fraction of reads can identify taxa (Table 1). However, amplicon sequencing has severe limitations, including low taxonomic resolution, copy number variations in rRNA genes [18], [19], as well as taxonomic biases related to using PCR amplifications [13], [14], [20]. Further, this technology can only inform the researcher of the taxonomy, but not the functions or genes present in a microbiota. To cope with this shortcoming, several bioinformatic algorithms were developed to infer the functional potential of communities based on taxa detected via amplicon sequencing, such as PiCrust2 [21] or FAPROTAX [22], but these predictions are inherently difficult to make without metagenomic data [23].

**Table 1:** Comparison of amplicon sequencing and shotgun metagenomics approaches.

Amplicon sequencing – Advantages	Metagenomics – Advantages
• Easy to use & cost-effective • Standardised approaches & mature bioinformatics • Clearly defined taxonomy • Good software for reference-free “species” delineation	• PCR free approach • Genomes of actual strains in sample can be assembled • MAGs can be the basis of or associated to GSMs • Very diverse analyses possible

Amplicon sequencing – Disadvantages	Metagenomics – Disadvantages
• Taxonomic biases in amplification • Resolution limited to genus or species level • Abundance estimates unreliable due to 16S/18S copy number variations and PCR biases • Actual gene content of species unknown • Taxonomic representation dependent on primer choice (e.g. Archaea require specific primers [13])	• Limitations imposed by sequencing depth, coverage requirements for successful assembly usually only met for dominant microbes • Complex bioinformatics & analysis • Still costly

#### 2.1.2 Metagenomics

The first forays into metagenomics were based on genomic fragments cloned into bacterial artificial chromosomes (BACs) [8], while later metagenomic approaches used random shotgun-sequencing of DNA molecules (e.g. marine seawater [24] or acid mine drainage [25]), befitting the definition of metagenomics as “*the application of modern genomics techniques to the study of communities of microbial organisms directly in their natural environments, bypassing the need for isolation and lab cultivation of individual species*” [26]. The acid mine drainage study is noteworthy, because this environment is so extreme that few microbes can survive here, with the two most abundant species being a) undescribed and b) at 75% and 10% abundance [25]. This enabled the *de novo* assembly of microbes not represented in databases, being the first example of a MAG and, in hindsight, predicting the future of the metagenomic field. Only 8 years later, metagenomic binning was performed again on a low complexity community, using a combination of biased DNA extractions, to create differential abundance profiles of the same organisms in the same sample. Bioinformatic binning was performed by clustering contigs based on their GC content, k-mer profile and abundance similarity [27], measures that have been used since then, with slight variations, in metagenomic binning.

#### 2.1.3 Importance of metagenomics for future projects

Reconstructing genes and genomes of microbes is extremely important to understand ecosystems, and fine-scaled deviations that might occur in non-normal states, such as complex diseases in hosts. This is because ongoing genome sequencing projects have shown the bacterial genome to be highly dynamic [28], with mobile genetic elements and other molecular mechanisms exchanging genes between strains of the same species, or between different species. Whether this genome fluidity is an adaptive mechanism is still under active debate [29], [30], but for diseases such as cystic fibrosis we know that pathogens lose virulence factors as an adaptation for long term host colonisation [31]. With newer data such as this, it is becoming clearer that key microbes in a microbiota, such as pathogens, should be identified by strain and not simply by their species membership. For example, *Escherichia coli* is a common commensal of the human gut, but some strains can be associated with pathogenic states including necrotizing enterocolitis in infants [32], cancer [33] or diarrhoea [34]. *Prevotella copri* strains have been metagenomically associated with specific metabolic niches [35]. Symbionts of deep-sea mussels were shown to offer distinct ecological metabolic functions to their host, despite having 100% identical 16S sequences [36]. Therefore, the pangenome-derived functional repertoire of a species cannot be captured using amplicon sequencing, but only using approaches such as metagenomics.

The *core metagenome* of a microenvironment likely contains mostly the core genomes of abundant species, essential to the survival of the species and thus likely mostly representing conserved core functions. This is in contrast to the *accessory genome* of a species, encoding more “exotic” metabolic functions used in more specialised circumstances and in response to local adaptations, local symbiosis or changing environmental conditions, functions that, hypothetically, represent interesting ‘edge-cases’, such as in host diseases. Here lies the potential of metagenomics, going beyond functional core predictions that could be inferred from amplicon sequencing [23], but instead cataloguing pangenomes that can be patient-, disease- or cohort-specific.

### 2.2 Metagenomic assembly & binning

Metagenomic assemblies of more complex microbial communities were initially assumed to be too complex for short-read based assemblers (see review of their evolution [37]), but in 2010 the MetaHIT consortium demonstrated this possibility using the specifically adapted SOAPdenovo assembler [38]. Gene predictions from metagenomic assemblies from the human gut established a “gene catalogue” that represents most of the non-redundant genes found in these metagenomes (clustered at 95% identity) [38]. Combined with mapping of metagenomic reads onto such a gene catalogue, the abundance (and presence) of the genes in each metagenomic sample can be estimated. Therefore, this method represents probably the purest incarnation of the ‘*bag-of-genes*' approach towards metagenomics. These genes can subsequently be used to determine species abundance and functional composition of metagenomic samples, by associating each to a given taxon or functional annotation (e.g. [39], [40]). However, these genes can also be clustered together dependent of the species’ genome they originate from. This was accomplished in 2014, when the human gut gene catalogue was binned into 741 metagenomic species (MGS), and subsequently used to aid in the assembly of MAGs from single samples [41]. The important technical advancement was the clustering of genes based on their abundance/occurrence across different samples using a computationally efficient canopy clustering algorithm, to cluster millions of genes into groups of co-occurring genes (Fig. 1). This first application of MAGs to human gut metagenomes showed an enormous taxonomic diversity that was otherwise largely unexplored, as only 17.4% (129/741) of identified MGS could be assigned to a sequenced species.

In the following years, metagenomic binning algorithms underwent several evolutions, essentially relying on better clustering approaches of metagenomic-assembled contigs and their abundance profile, GC content and k-mer content. These approaches were implemented in pipelines such as MetaBAT2 and MaxBin2 [42], [43], complemented by tools to determine the quality of binned genomes like CheckM [44] (reviewed in more detail in [45]). These advances in metagenomic binning led to a much better understanding of microbial communities over the past five years, such as the discovery of hundreds of novel taxa from diverse microbiomes [46], the discovery of new Archaeal branches in deep ocean samples [47], and the addition of thousands of novel taxa of the human gut microbiome in 2019 [48], [49], [50].

It should be noted that authors of these studies usually highlight more complete mapping of metagenomic reads following inclusion of new references, such as with gene catalogues [38], [51] or reference genomes for the human gut [41], [50]. These improved databases are essential for reference-based taxonomic profiling of metagenomes (e.g. MetaPhlAn2, mOTUs2 and Kraken2 [52], [53], [54]). Indeed, reference-based taxonomic profiling approaches have a much better computational performance, are straightforward to use and do not require much expert knowledge compared to *de novo* taxonomic profiling, which is instead completely reference-independent and relies on *de novo* assembled metagenomes, constructed gene catalogues or MAGs. Despite these drawbacks, we argue that the *de novo* metagenomic approaches should become the standard in microbiome research, because the experience of the past decade has shown an ever-expanding microbial diversity; diversity often not captured in reference-based approaches. For example, in antibiotic-treated patients, we found a community entirely dominated by a novel order of bacteria [55], which was entirely missed with reference-based approaches because the novel species was too distinct from known bacteria in reference databases. This problem is only exponentially amplified in microbiotas that are less well characterised, like in the soil [5]. Lastly, to move beyond a ‘*bag-of-genes*' approach, or entirely ignoring the metabolism happening in these environments, MAGs and the gene functions associated to these are important building blocks in what is considered next-generation metagenomics.

### 2.3 Functional annotation of bacterial genomes

Extensive publicly available databases of reference genomes provide a rich background for comparing microbial genomes. Among these are NCBI (www.ncbi.nlm.nih.gov), JGI’s IMG/M [56] and proGenomes2 [57] that host each 241 993, 80 295 and 87 920 prokaryotic genomes as of March 2020, respectively. All of these draft genomes are curated to different degrees in the databases with automatic algorithms, greatly advancing the availability and ease-of-use for reference genomes. Yet, functional annotations of predicted genes remain limited, with usually less than half of a genome being functionally annotated. It says something of our ability to annotate genomes, that the proportion of a genome functionally annotated is often correlated to the genetic distance to the very well researched *Escherichia coli* (anecdotal observation). However, progress has been made and several databases exist today that can be used to infer functions assigned to taxa, some focusing on giving broad spectrum functional annotations, others on specialised functions such as transporters.

Broadly used resources relevant for functional annotation are for example KEGG [58], UniProt [59] or eggNOG [60], each having different goals. KEGG (Kyoto Encyclopedia of Genes and Genomes) relies mostly on well-annotated reference organisms, hence it offers well-annotated metabolic pathways and modules. However, KEGG Kyoto Encyclopedia of Genes and Genomes was initially conceived for the description of eukaryotic pathways, and is of limited taxonomic range. UniProt (Universal Protein resource) is a protein database, offering different layers of information, such as functional annotations, subcellular location, catalytic activities, protein–protein interactions, variants or protein structures. It is organised into four main databases that differ in their level of expert curation, literature mining and computational annotations. eggNOG (evolutionary genealogy of genes: Non-supervised Orthologous Groups) is largely developed from computer-based predictions, but offers wide taxonomic range. It is not organised in metabolic pathways and functions. The underlying principle of eggNOG is to calculate clusters of orthologous groups [61] among prokaryotic and eukaryotic reference genomes. This evolutionary approach is powerful, because orthologous genes are functionally stable, occasionally even between different species [62]. These represent major taxonomic groups in the eggNOG database that allow for taxonomic annotations even in novel genera or families, such as implemented in the program eggNOG-mapper [63].

In addition to these general resources, many specialised databases and specialised algorithms were developed to annotate genes in specialised functions, such as antibiotic resistance (see review [64]), transporters (TCDB, [65]), or carbohydrate active enzymes (CAZy, [66]), both important in interpreting the metabolic capabilities of a microorganisms, as is also the goal for genome scale metabolic models.

## 3 Genome-scale metabolic models

Genome-scale metabolic models (GSMs) describe the metabolic network of an organism and its interaction with the environment based on the enzymes encoded by the genome. Contrary to the small-scale pathway-specific models, it enables us to investigate systems-level metabolic properties and functions, and identify a mechanistic link between cellular genotypes and metabolic phenotypes through gene-protein-reaction (GPR) associations. Therefore, combined with metagenomic approaches, GSMs provide a way to delve into the functional potential carried by a genome. Mathematical models applied to GSMs address questions on the physiology of the organism and considering the interactions between GSMs is an effective means of characterising communities and microbiota.

### 3.1 Reconstruction and analysis of GSMs

• Draft reconstruction

The reconstruction of good-quality GSMs is an iterative process. Initial draft reconstruction is fairly straightforward: involving the extraction of reaction stoichiometries and reversibility from the organism specific biochemical databases, such as BioCyc [67], [68], KEGG [69], [58], BIGG [70], [71], and BRENDA [72] or use of tools, such as Pathway Tools [73], [74], ModelSEED [75], KBase [76] and CarveMe [77], that take either a sequence or an annotated genome to automatically reconstruct the draft model. However, these automatically-constructed draft models can be of poor quality with inconsistent naming of metabolites and reaction identifiers, incorrect reaction stoichiometries and reversibility, missing or incorrect GPR associations and gaps in the metabolic network [78]**.** Thus, to obtain a realistic metabolic representation of an organism, these draft metabolic models need to be refined/curated by expert users, as described in the following sections.

• Definition of externals and transporters

Usually the large-scale metabolic models such as GSMs are analysed based on the law of mass conservation. The conservation principle requires the assessment of all inputs, being metabolites that are taken up by cell from the extracellular environment such as substrates present in the growth media, and outputs, being terminal metabolic products that are exported out of the system. These metabolites are regarded as *external metabolites*’ or *boundary metabolites*’. *Internal metabolites*’, on the other hand, do not interact with the environment and are balanced with respect to production and consumption in a steady state [79], representing a class of functions that in the ‘*bag-of-genes*' metabolic model would lead to incomplete assumptions. The *transport reactions*’ or *exchange reaction’*, inter-convert the internal and external metabolites and thus, connect the environment to the metabolic system. However, the annotation of transporters is still a bottleneck. Under such circumstances, tools to predict transporters from genomes can be applied [80], [65]. In addition, growth experiments on defined media can be used to infer the presence of transporters for the import of external substrates. The output of the system is generally defined in terms of excreted by-products and cellular biomass products. The latter is formulated by defining the fractional contribution of the macromolecular content of the cell, such as the fraction of lipid, protein, RNA, DNA, and the metabolites that make up each macromolecular group such as amino acids and nucleotides [81].

• Curation and validation

Curation of GSMs is usually an iterative process where individual reactions are ensured to be atomically balanced, names of identifiers are made consistent, reaction reversibility is corrected so that the model is not able to generate energy or mass in the absence of relevant substrate import [82], and missing reactions are identified for “gap-filling”, with the help of a wide range of tools [83], [84], [85]. Experimental data such as growth on defined media and biomass composition are used for gap-filling and refinement of the network which often lead to updated GPR associations.

The curated model, thus ensures the laws of mass and energy conservation, is free from stoichiometric inconsistencies [86] and is able to produce biomass components in experimentally observed proportions from the defined media known to support the growth.

• Analysis

GSMs are analysed mainly using linear programming (LP) based optimisation techniques, commonly Flux Balance Analysis (FBA) [87], [88], [89], based on the law of mass conservation. It assigns fluxes to reactions for a given objective function and set of defined constraints assuming that the system is at steady-state. The most typical objective functions are maximisation of growth rate, and minimisation of total flux as a proxy for economy of investment in enzymatic machinery. The constraints are used to apply upper or lower limits on reactions, and export of one or more products/biomass while the steady-state assumption implies that the rates of formation of internal metabolites is equal to the rates of utilisation and thus, concentration of metabolites remain constant over time. Other approaches to model producibility and activation of reactions exist, including the use of the network expansion algorithm [132].

### 3.2 Pitfalls of genome-scale metabolism reconstruction

Since the publication of the first GSM in 1999 [90], thousands of GSMs have been reconstructed (either manually or using automated tools) and made available to the community in the previously cited databases. They concern diverse organisms belonging to Bacteria, Archaea, and Eukaryotes. They have been useful in analysing cellular behaviour under different genetic and environmental conditions, designing defined growth media and drug targets, and investigating metabolic interactions in microbial communities (reviewed in [91], [92], [93], [94]). However, there are still limitations in the field.

The foremost limitation includes the requirement for manual intervention and curation. As detailed in the above section, there are metabolic modelling tools that supports the automated construction of GSMs however, the need for extensive manual curation has not yet been fully replaced by these automated methods which includes defining transporters, biomass components and gap-filling [95], [96]. Though to the certain extent, gap-filling, has been accounted for in metabolic modelling tools, this usually can result in over-fitting of the network and thus, needs careful examination by the user [96]. On the other hand, although the efficacy of GSMs is linked to the accuracy of the biomass composition [81], [97], due to the lack of species-specific experimental data most GSMs still rely on the biomass composition available for a handful of model organisms, e.g. *E. coli* [98], [99].

Further limitations include the underlying assumptions and choice of objective functions that are used to guide the reconstruction and the refinement of the model. The steady-state assumption makes the computational analysis of large-scale metabolic models possible by restricting the space of possible solutions. However, this also leads to loss of information on dynamic behaviour and possible accumulation of metabolites in the system. Additionally, widely used LP-based optimisation techniques treat reaction fluxes as a variable, though most of the enzymatic reactions are indeed a function of metabolite and enzyme concentrations, and enzymatic property, and do not account for the kinetic properties of the network. Objective functions that vary around maximisation of demand or minimisation of cost have been widely used for GSMs analysis and proven to be useful [97], [98], [99]. However, the biologically relevant question remains: “can we apprehend the objective of a living organism/cell under given conditions?”. Currently, there is no easy answer to this broad topic, with this remaining to be explored theoretically, experimentally and computationally.

It also has to be noted that genome-based GSMs present the putative metabolism of an organism, ignoring the fact that genes coding for enzymatic reactions are not necessarily expressed, and that their expression can vary with time and the environmental conditions. The incorporation of omics data, such as transcriptomics and/or proteomics, and transcriptional regulatory information has been shown to improve phenotype simulations obtained from GSMs [100], [101], [102], [103], [104], [105]. Likewise, the human metabolic network can take into account tissue/organ-specific information and metabolic models can be obtained for tissue/organs or tissues of interest [106], [107], [108], [109]. Metabolic models of intestinal cells [106], [110], [111] can be beneficial resources to model host-microbiota interactions [112].

Despite above mentioned limitations, metabolic models constitute a good proxy of the functional potential carried by a genome. A large community of researchers are involved in the development and improvement of methods for their reconstruction and subsequent analyses. Some limitations remain but the reconstruction of GSMs is now systematic when studying an organism. This is notably due to the efforts in providing automatic methods for the inference of GSMs from genomes. Curation is still needed but automatically-reconstructed drafts are already informative. This is an asset for the use of GSMs in metagenomic studies, that are required to scale the reconstruction and subsequent treatments of GSMs to hundreds or thousands of genomes. The application of GSMs to the study of communities of organisms has led to many developments in the last decade, some of which we will review in the next section.

## 4 Community approaches of metabolism modelling

Organisms exhibit complex interactions with both other organisms (biotic interactions) and their natural environment (abiotic interactions). The former notably permits to account for auxotrophies, for example for amino-acids or vitamins [113], and limited nutrient availability in environments. A wide range of these interactions are of metabolic nature, from the exchange of metabolites in cooperative events, to the competition for limited nutrients in a niche occupied by microorganisms. From a systems perspective, metabolic modelling is therefore particularly suited to analyse microbial communities. Comparing, analysing and integrating this ‘*bag-of-genomes*’ approach can help in understanding the complex interactions that govern the assembly and evolution of microbiotas: abundance of all microbes, their growth rates, the composition of the environment, the metabolic state of each member… Yet, limitations that apply to the modelling of single organisms persist when scaling this to communities, turning this topic into a field with a constant need for development. Methodologies applied to the study of communities are highly diverse and a classification can be established in the context of metagenomics. Broadly, one needs to distinguish between bottom-up and top-down approaches. Bottom-up approaches rely on existing work on individual metabolic models of microbes to infer interactions between these organisms, when considered living in a shared environment. They are mainly applied to small communities for which experimentation is manageable, with culturable micro-organisms or high-quality metabolic models that are already available. In contrast, top-down approaches rely on the identification of microorganisms using metagenomic data and subsequently exploiting methods for metabolic modelling.

**Fig. 2:**
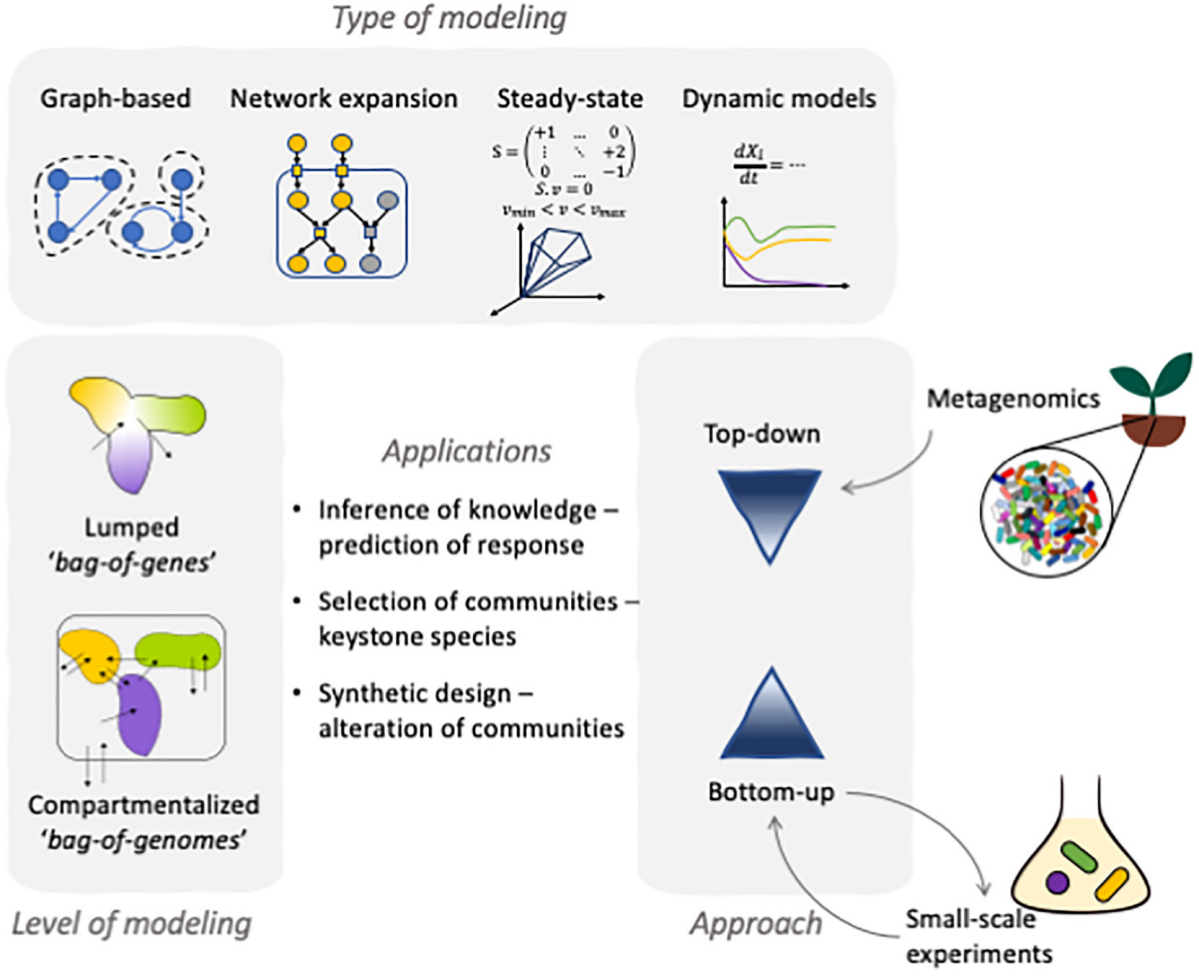
Diversity of methods for metabolic modelling in communities of organisms.

There are a variety of questions to address, ranging from the understanding how a microbiota is assembled from its single units (e.g. recovery of microbiomes after antibiotic treatments), to active interventions that alter its state (e.g. pre- or probiotic interventions) or *de novo* building of a community with desired features (e.g. for industrial purposes) (Fig. 2). A first matter is the identification of interacting species. While this can be addressed with co-occurrence analysis [114] or correlations [115]; yet, these methods do not give insights into the mechanistic nature of the interaction [116]. For example, the authors of [117] associated 16S rDNA sequencing to metabolic models to identify pairwise interactions that were classified as positive or negative. More generally, pairwise interactions are often discretised according to the beneficial or detrimental effect for each member of the pair. Among interactions, mutualism is beneficial for both, leading to cooperation events, whereas competition is detrimental for partners [118]. Interactions can be beneficial for a single partner, leading to commensalism (respectively parasitism) if it is neutral (respectively detrimental) for the second partner. Interaction networks are only a first step of analysis, as understanding communities is dependent on understanding the mechanisms of these interactions.

Secondly, metabolic modelling can help in getting insights into the nature and mechanisms of microbial interactions, and provide predictions on the metabolic dependencies between species. In [119], the authors combine models and cocultures of gut bacteria to identify positive and negative pairwise interactions. Using *exo*-metabolomics on monospecies, they characterised the utilisation and secretion of metabolites thereby composing putative competition and interchange networks. With more species-rich communities, these predictions become more computationally intensive, as the number of interactions scales exponentially to species richness: authors of [120] made over two million simulations of two-species communities. This was used to assess the potential of costless secretions of metabolites as a driver for interactions, concluding on its positive effect on the stability of communities.

Thirdly, facing the complexity of microbial interactions can be contemplated through community reduction to identify keystone species that enable new emergent properties of communities [121], [122], such as *Methanobrevibacter smithii* [123] in the gut microbiota. In the same direction, metabolic modelling is applied to the synthetic design or bio-engineering of communities [124], [125]. A final goal is to develop methods for the perturbation and modulation of the microbiota towards a defined objective [3]. In the next sections, we will present the strategies used by modellers to address these questions.

### 4.1 Metabolic modelling techniques in communities

The main questions and objectives above rely on diverse methodologies. A first distinction is the possibility to consider a *lumped* or a *compartmentalised* model of the microbiota [126], [127]. In the former case, an asset is to access the functions catalysed by microbes directly from a metagenome, or by merging metabolic models [121], [128], [129] which corresponds to modelling the metabolism of the ‘*bag-of-genes*'. However, methods using lumped models do not enable the identification of the role of each member of the community. This is addressed by compartmentalised models.

In a similar way to individual organisms, several semantics and techniques can be applied to communities of organisms; these being summarised in Fig. 2.

• Network-based

Network or topology-based analysis can provide insights into the metabolism of an interacting community. By applying metrics and comparing the contents of metabolic networks in terms of reactions, it is possible to assess a potential for cooperation if the metabolic inputs of one partner are present in the metabolism of the second, or competition, if both partners share metabolic inputs. NetSeed is a tool to compute such inputs for individual metabolic networks [130]. NetCooperate relies on such concept to compute two scores: the metabolic complementarity index and the biosynthetic support score [131].

• Graph-based

Network expansion is the graph-based modelling of producibility in metabolic networks introduced by [132] which can also be extended to communities [133]. Kreimer et al combine network expansion and NetSeed to calculate the effective metabolic overlap as a proxy for the competitive potential between two bacterial species [134]. Ofaim et al also apply both methodologies to the metabolic modelling from gene catalogues of several environments [129]. Such methodology has been applied to bacterial communities of the insect *Wolbachia* after reconstruction of their respective metabolic networks based on genomic information [135]. In [122], we introduced MiSCoTo to select minimal communities in large microbiotas. It combines the network expansion algorithm to two optimisation problems aiming at minimising the number of interacting species (lumped model/’*bag-of-genes*') and/or the number of needed metabolic exchanges (compartmentalised model/’*bag-of-genomes*') for a desired function. Graph-based models of communities are also used in MultiPus [124] for the synthetic design of consortia.

• Dynamic modelling

Using longitudinal data, it becomes possible to tackle the evolution of the composition of communities in a microbiota. Ordinary Differential Equations (ODE) models such as the consumer-resource or the generalised Lotka-Volterra (gLV) have been used. They are often applied to amplicon sequencing longitudinal data. Goldford and colleagues have monitored the assembly and stabilisation of diverse natural communities in a controlled environment [136]. They observed convergence of the microbial composition to a family-level attractor for communities that were initially very diverse. By varying the carbon source available for the consortium, they noted at the end of the experiments a greater similarity between communities grown on similar nutrients than between communities originating from the same environmental source. This suggests that the fate of the communities in terms of composition was mainly driven by the availability of nutrients, which can be explained by a generic consumer-resource model. In addition, gLV models have been extensively applied to study the temporal evolution of communities [119], [137], [138], [139], [140]. These models enable predictions of community dynamics by taking into account growth rates and interactions strengths between microbes [141].

• Steady-state modelling

Studying the precise dynamics of metabolic systems can be done with kinetic models relying on Michaelis-Menten equations. However, this requires the identification of kinetic parameters of enzymes, which is not experimentally conceivable for all reactions of all organisms. [142] integrated the kinetic model of the core metabolisms of *Escherichia coli* to its genome-scale metabolic model. When considering communities of organisms, a common practice is to rely on the steady-state assumption (no accumulation of internal metabolites) and apply constraint-based models. The first multi-species stoichiometric model of metabolism was focused on *Desulfovibrio vulgaris* and *Methanococcus maripaludis* [143]. This work paved the way for many applications, including the design of growth media ensuring a desired interaction type between species [144] or reducing the cost for metabolic cooperation [145]. See the work of [146] detailed applications of GSMs to medium design. Several bottom-up methods are dedicated to assemble existing metabolic models in order to predict the interactions and evolution of a community composed of the corresponding microbes. OptCom [147] takes into account an individual’s biomass to be maximised for each microbe in an inner problem, and an outer problem consisting in a community-level fitness objective. Budinich *et al* explores the pareto front of a multi-objective optimisation of a three-member community [148]. SteadyCom [149] infers flux distributions in the steady-state model of a community across time. In their work introducing the CASINO Toolbox, Shoaie *et al* first initialise the community at the level of individual species, then optimise resource distributions within all partners [150]. An approach that we could qualify as top-down in the context of microbiota exploration is the one of MMinte [117] that matches the 16S rDNA sequences of a sample to complete genomes of NCBI and reconstruct metabolic models for these genomes using ModelSEED [151]. FBA is then run on individual GSM or pairs of GSMs and pairwise interactions are predicted.

Temporality is a crucial parameter when studying communities, ODE can be associated to steady-state models in order to take kinetic parameters into account. Dynamic FBA (dFBA) has been applied to communities, thereby developing the framework of Dynamic Multi-species Metabolic Modelling (DMMM) [152]. dOptCom extend the multi-objective simulation of communities of OptCom to capture kinetics information [153].

Finally, it is important to note that the spatial arrangement of microbes matters for interactions. COMET [154] and BacArena [155] are two methods that take spatio-temporal parameters into consideration.

### 4.2 Pitfalls of GSM in community modelling

Difficulties associated with the reconstruction of individual metabolic models also apply to their assembly into communities. The latter has additional limitations, brought by scalability issues and the limited control over the studied environments for validation of hypotheses. The quality of the individual reconstructions is crucial for quantitative and temporal simulations [79]. Particular attention has to be given to the characterisation of transport reactions. For automatically-reconstructed metabolic networks with poorly-characterised transporters, it is possible to suggest exchanges as mechanistic hypotheses for cooperation when proposing minimal communities [122]. Such predictions are provided exist through in a reduction of the highly combinatorial search space of interactions, and have to be treated as such and *a posteriori* filtered.

A main pitfall in GSM reconstruction concerns non-model organisms including those that cannot be grown in single cultures. In metagenomics, typically most species cannot be cultured and/or the community is too complex for extensive culturing experiments. Here, the risk is to overfit community GSMs with respect to an objective (e.g. biomass reaction) with reactions from the gap-filling step [146], [156]. To address this, several strategies exist and gap-filling can therefore be performed either before or after the creation of the consortium of models [157]. On the contrary, the authors of the work presented in [156] used drafts of metabolic networks without performing the gap-filling step. This is relevant to avoid adding false positive reactions that could hide a need for metabolic cooperation between organisms.

The complexity of communities drives constant adjustment of the metabolic composition of the environment. Models set up an initial composition of the medium which will be modified by the secreted molecules of each microbe. However, precisely assessing the metabolic composition of a microbiota is a difficult task. Using metabolic network percolation, the authors of [156] designed a probabilistic approach for deciphering whether metabolites are likely to be produced by the metabolism of an organism or retrieved from the environment.

Finally, in the methods presented above, several strategies are used to design the objectives for optimisation. They can consist in a combination of individual and community objectives. The optimisation of growth that is generally the basis of GSM reconstruction is also frequently found in communities. Selecting an objective function or a combination of several functions with biological relevance for the considered community is a complex task completed only by considering biological and evolutionary knowledge.

### 4.3 Top-down approaches suitable to large metagenomes

Most approaches that model communities with metabolic networks focus on a small number of members and highly-curated GSMs. When using culturable organisms, it is advisable to sequence their genomes, or use the genome of closely related strains from genome databases, to *de novo* reconstruct GSMs. Another possibility is to use curated GSMs from databases and simulate the community with adequate environmental settings, but strain-specific genome differences likely exist [158] and the proper strain-specific integration of GSMs into metagenomes is still an active field of research. In the gut microbiota, 818 curated GSMs are available for simulation (AGORA) [159], together with diet information and the human metabolism [160], constituting a remarkable resource for tests and validations of algorithms. As an example, Diener et al designed MICOM, a metabolic model of the human gut microbiome [161]. Starting from shotgun metagenomics, abundance profiles for taxa were calculated and representatives of taxa were identified within the AGORA GSMs. A majority of genera found in metagenomic samples could be mapped to available GSM reconstructions, although the more precise the taxonomy (species, strain), the fewer number of available representatives. MICOM was also applied to the characterisation of hydrogen sulphide production in microbes in the context of colorectal cancer [162]: 16S rDNA sequences were aligned to complete genomes from which draft GSMs were derived using PATRIC [163]. The approach used by MMinte [117] starts with metataxonomics and automatically constructs *de novo* GSMs from available, closely related species. However, a proportion of OTUs will not be mapped to genomes and this part of the metabolism is not taken into account. To address such problems, Greenblum *et al* built a single lumped metabolic network from a ‘*bag-of-genes*' approach without a priori assembly of the individual genomes [128]. This enabled modellers to retrieve a large spectrum of functions but their assignment to individual microbes, and therefore compartmentalisation and modelling as ‘*bag-of-genomes'*, is missing. Table 2 summarises a number of tools and frameworks for community modelling.

**Table 2:** Comparison of some tools and frameworks for GSM-based modelling of interactions in communities. BU: 'bottom-up' i.e. association of individual GSMs into small communities. TD: 'top-down' i.e. analyses starting from large metagenomic-identified communities.

Tool/Framework	Modelling	Application	Approach
DMMM [152]	dynamic steady-state	a community of 2 bacteria	BU
OptCom [147]	steady-state	multi-objective optimisation of communities from 2 to 4 species	BU
dOptCom [153]	dynamic steady-state	multi-objective & multi-level optimisation of 3-species communities	BU
CASINO [170]	steady-state	6-species communities	BU
COMETS [154]	dynamic steady-state + spatial	2 and 3-species communities	BU
BacArena [155]	dynamic steady-state + spatial	7-species community	BU
SteadyCom [149]	steady-state	4 and 9-species communities	BU
Greenblum *et al* 2012 [128]	topological	‘bag-of-genes' per sample	TD
Metage2Metabo [164]	network expansion	de novo GSM reconstruction, global analyses and community reduction	TD
MMinte [117]	steady-state	pairwise analyses and interactions	TD
MICOM [161]	steady-state	metagenomic samples mapped to existing GSMs or newly reconstructed GSMs drafts from genomes following OTUs alignment	TD

In an era where shotgun metagenomics provides sequences suitable for MAGs reconstruction and the methods for such reconstruction are rapidly improving, it appears relevant to bridge the gap between MAGs and metabolism, allowing us to study (a part of) the functions of these often-uncultivated species. Yet, approaches relying directly on MAGs for metabolic network reconstructions are lacking. Continued attempts are being made to alleviate this problem, notably through the implementation of large-scale and parallel automatic reconstruction with Pathway Tools in [164]. This method is directly applicable to MAGs and the gap-filling step is not performed to prevent missing interactions. However, because of the lack of manual curation, comparisons are missing when assessing the part of the metabolism that is inevitably lost when working with MAGs and automatic inference of metabolism.

As depicted in Fig. 1, several analytical paths exist to model the metabolism of large communities at the genome scale. Starting from amplicon sequencing and OTUs, two solutions can be contemplated. The first one is to target existing, ideally curated, GSMs for close species/strains of the OTUs [165], [166], [167], [168]. For the intestinal microbiota, AGORA already provides a large number of GSMs for community simulations. Despite this, it is a considerable challenge to choose a well-fitting GSM, depending on proximity between the 16S rDNA genes of both species, as often OTUs can represent genera or families that are not well represented in GSMs databases. The second possibility is to identify close genomes for each OTU available in public genome databases, and subsequently *de novo* build draft GSMs from these genomes [162], [117]. The obtained GSMs will undoubtedly be of lower quality due to the lack of manual curation, but one asset of the method is to target genomes that are closer to the OTU. A common problem is that functions in the resulting GSMs can differ from the actual functions in the community, as it is well documented that even between strains substantial differences exist of member from the same species [28]. Therefore, it might be the safer choice to follow an analysis of community GSMs, starting from MAGs obtained via shotgun metagenomics (Fig. 2). While this is limited by potentially incomplete or chimeric MAGs, and missing manual GSM curation, this path will very likely become more prevalent in the next few years, as the availability of MAGs reconstructions increases. The asset of such methodology is to free the modelling procedure from the availability of genomes or GSMs in databases, obtain host-specific GSMs (automatically constructed) and understand the possible metabolic role of unknown genera, not represented by cultured GSMs.

Currently one obstacle to overcome is that few top-down approaches are able to meet the demand of larger metagenomic studies, often in the scale of hundreds or thousands of species’ metabolic networks and potential interactions. To address such limitation, the use of coarse-grained models have been suggested for the inference of mechanistic information from experimental data on communities [169]. Several levels of granularity can be envisaged, such as summarising a group of reactions into pathways or groups of functions. This can be a solution to the incompleteness of MAGs that can lead to missing reactions. An alternative resides in the use of more scalable techniques such as a topological semantics to represent producibility [122] or a network flow-like approach [121]. A general objective of these tools is to identify species or minimal communities of interest and thereby reduce the number of partners to consider, in order to enable the use of more precise and quantitative simulation methods. Tackling these limitations will see one important step in better defining the characteristics of the keystone species, and to selecting these with higher precision.

## 5 Summary and outlook

Metabolic modelling of microorganisms has substantially improved the knowledge on mechanisms involved in interactions within communities. Scaling the ‘*bag-of-genomes*' models to large communities to capture the whole diversity of functions in a microbiota remains challenging. Ensuring the good quality of GSM reconstruction from automatic methods is crucial as manual curation and manually-guided reconstruction of metabolism is not conceivable for thousands of genomes. Another alternative to consider is to develop formalisms for metabolic modelling that are robust to missing genes and functions, therefore still enabling the inference of relevant interactions between microorganisms of an ecosystem. The field of metabolic modelling in large scale communities is hypothesis-driven, aiming to propose subcommunities, interactions and organisational models of ecosystems. The search space for these questions is large due to the high combinatorics brought by the number of members in the microbiota. This motivates the use of optimisation problems to constrain and reduce the search space in adequation with the knowledge in microbial systems ecology. However, as it is difficult to combine experimentations and predictions, especially for large-scale microbiotas, validation of interaction prediction remains a bottleneck that needs to be addressed in the next few years.

The ongoing revolution in *de novo* reconstruction of genomes from shotgun metagenomics (MAGs) enables a more precise characterisation of complex microbial communities and their emergent properties, with applications in all domains of life sciences. However, as these genomic steps form the basis for all subsequent analyses including the functional characterisation of organisms, it is crucial for the MAGs to be reliable and to reflect the diversity of the sampled environment. In this direction, strain-resolved metagenomics appears as a critical open challenge in the field, and given the extraordinary genomic plasticity of different strains of the same species [28], it becomes all the more important to not only define a core genome of a species, but to capture reliably the pangenome. Therefore, specialised functionality of a strain residing within a patient can be characterised together with the metabolic pathways expected within a community, to enable personalised microbiota profiling. We propose that these auxiliary functions might be the key to understanding complex diseases, with metabolic functions that are less frequent in “normal” microbiomes. To apply this principle to host-associated microbiomes and complex diseases, these functions could represent either host-derived metabolites in abundance due to a disease, or metabolites that contribute to, or trigger, a disease in the host. Using only taxonomic information or GSMs from reference species, one would overlook such deviations. Therefore, there is an urgent need for the metagenomic field to model and understand bacterial communities beyond the taxonomic level. This will for example enable to accurately model person-specific gut communities, with major implications to understand patient-specific differences in food and drug metabolisation. We predict that efforts in this direction will lead to *in silico* predictions of the emergent metabolic capacities in environmental and host-associated microbiotas, leading to a better understanding of ecosystem deteriorations and complex diseases.

## CRediT authorship contribution statement

**Clémence Frioux:** Visualization, Conceptualization. **Dipali Singh:** . **Tamas Korcsmaros:** . **Falk Hildebrand:** Conceptualization, Visualization.

## Declaration of Competing Interest

The authors declare that they have no known competing financial interests or personal relationships that could have appeared to influence the work reported in this paper.
